# Managers’ experience of causes and prevention of sick leave among young employees with Common Mental Disorders (CMDs)–A qualitative interview study with a gender perspective

**DOI:** 10.1371/journal.pone.0292109

**Published:** 2023-09-27

**Authors:** Helena Tinnerholm Ljungberg, Caroline Olsson, Irene Jensen, Lotta Nybergh, Elisabeth Björk Brämberg

**Affiliations:** Unit of Intervention and Implementation Research for Worker Health, Institute of Environmental Medicine, Karolinska Institutet, Stockholm, Sweden; University of KwaZulu-Natal College of Health Sciences, SOUTH AFRICA

## Abstract

**Background:**

Young adults entering the workforce have an almost 40% greater risk of work-related mental health problems than other working age groups. Common mental disorders (CMDs) constitute the majority of such mental health problems. Managers are crucial in promoting a good psychosocial work environment and preventing sick leave. The study aims to explore managers’ experience of 1) causes of sick leave in the personal and work-life of young employees with CMDs, and 2) prevention of such sick leave. A gender perspective is applied to examine managers’ experience of causes and prevention of sick leave in relation to male and female employees and male and female-dominated occupations.

**Material and methods:**

A qualitative design was applied and 23 semi-structured interviews were conducted with Swedish managers experienced in supervising young employees with CMDs. The interviews were analysed with conventional content analysis and the managers’ experience of similarities and differences between young female and male employees and occupations were explored through reflective notes.

**Results:**

Four main categories and eight subcategories describe the managers’ experience of the causes of sick leave due to CMD among young employees. The main categories are: 1) entering work life when already worn-out, 2) struggling with too high expectations at work, 3) having a challenging personal life, and 4) being unable to manage specific occupational challenges and demands. Gender differences were found in six subcategories regarding, e.g., work demands and problems in personal relationships. One main category and three subcategories describe how this type of sick leave might be prevented, with managers emphasizing the need to ease the transition into work life. Gender differences in the prevention of sick leave were found in one subcategory regarding communication about workers’ health and problems at work.

**Conclusion:**

Our findings show that gender norms and the expectations of young men and women are factors of importance in managers’ experience of the development and prevention of CMDs. These results can inform their preventive work and their supervision and introduction of newly-employed young adults.

## Introduction

Common mental disorders (CMDs), i.e., depression, anxiety, adjustment disorders and stress-related ill health, constitute a large part of all mental health conditions known to cause suffering, risk of isolation, and stigmatization. CMDs also entail economic loss for individuals and societies in most Western countries, not least due to the sick leave that they may cause [1]. It was estimated in 2019 that an average of one in six young adults (15–29 years) in the EU had a mental health issue [2]. Given the high prevalence of CMDs and their consequences for the individual, the labour force and society, it has been argued that workplaces and managers play an important part in preventing sick leave by promoting workplace health and identifying risk situations [1].

Young adults entering the workforce have been described as a vulnerable group with an almost 40% greater risk of work-related mental health problems than other working age groups [3]. Possible explanations for the increased risk include the fact that these individuals lack previous experience of working life, while at the same time they are in the process of transitioning to adulthood [3]. However, the group is not homogenous, and to get a better understanding of young employees’ risk of sick leave, researchers have emphasized that they need to be treated as “a heterogeneous group [whose] vulnerabilities to occupational safety and health risks are contextual” [4, p. 3]. This implies that research on young employees needs to shift away from focusing on single factors and instead include the intersections between, for example, age, gender, and various other social categorizations [5], it also needs to take variations in working situations into consideration. One crucial factor is occupational sector. For example, research shows that young employees in Sweden working in health care and education have a higher risk of sickness absence due to CMDs than do young employees in other sectors [6]. Moreover, almost every second young worker in Europe has a temporary contract, and it is common for young employees to have part-time or shift work, both of which are known to be work-related risk factors for mental ill health [3, 7, 8].

There is a gender gap in sick leave due to CMDs. In Sweden, 50 percent of all women’s sick leave is due to mental disorders, while the equivalent number for men is 39 percent [9]. Among young women and men (16–29 years), CMDs are an even more common cause of sick leave, and the gender difference remains about the same as in the total population [9]. However, the gender gap in sickness absence is still not fully explained [10]. Two commonly invoked explanations of gender differences in sick leave are “the exposure hypothesis,” which posits that women are more exposed to risk factors in their occupations than men due to horizontal gender segregation in the labour market, and “the vulnerability hypothesis,” which posits that women are more vulnerable than men to exposures and risks [11].

### Managers’ roles and responsibilities pertaining to CMDs and work

In Sweden, employers bear extensive legal responsibility for providing a safe and sound work environment and preventing work-related stress [12, 13]. This responsibility started with the Work Environment Act of 1977 [14]. In the EU and other European countries, the psychosocial aspects of work have been receiving greater interest recently, although Sweden is among the few EU member states that explicitly mention the assessment of psychosocial risks at the workplace in occupational safety and health (OSH) legislation [15].

An increasing amount of research has been directed at understanding employers’ and managers’ roles as stakeholders in the psychosocial work environment and employee mental health. Research has shown that managers’ leadership behaviors affect employee productivity, health, well-being, and safety at work. However, previous research has tended to prioritize measures of employee performance over measuring employee well-being [16]. One qualitative review found that several previous studies had focused on change-oriented leadership. It reported limited evidence of this leadership behavior having a positive effect on positive hedonic well-being among employees, typically operationalized as job satisfaction [16]. Nevertheless, managers’ view of and attitudes to sick leave due to CMDs have rarely been explored. A few studies have started to fill this gap, for example by addressing how managers understand the work capacity of employees with CMDs, and whether detection of early signs (such as an employee’s decreased work performance) could help managers act, increase support, and hopefully prevent sick leave [17]. Another study has shown that receiving education about CMDs, and working for an organization that supports and stresses the importance of CMD prevention, improves managers’ preventative work [18]. Research into the attitudes of male and female managers has concluded that male managers tend to have a more negative attitude toward employees with depression than do female managers [19], and that female managers report more often than their male counterparts that they work preventively with CMDs [18].

None of the studies mentioned above address the gendered aspects of young adults on sick leave due to CMDs, and there are, to the best of our knowledge, no studies investigating the role of managers in dealing with CMD among young employees. Our previous research on work, personal, and lifestyle-related factors among young employees with CMDs shows that they perceived the causes of their sick leave to be mostly work-life related [20]. The present study furthers our understanding by adding the views of managers with experience in supervising young employees on sick leave due to CMDs.

### Gender theoretical framework

This study applies a theoretical framework previously described in Olsson et al. [21] in which gender is seen as a permeating yet dynamic structure in society [22, 23]. One way this structure is upheld is through gendered norms and expectations that constrict and shape the lives of women and men in different ways. In general, the gendered order places men and male values/attributes above women and female values/attributes in a gendered hierarchy [23]. Feminist and gender researchers interested in gendered norms and expectations in work life have previously demonstrated pervasive gender inequalities between men and women in work life, and between male and female-dominated sectors and occupations. These include wages, work environment and conditions [24]. Gender researchers have also emphasized the importance of other social classifications, such as class, ethnicity and age, and the intersection of those classifications with gender, when analysing the complexity of work inequalities [25] and health [26]. Like gender, these social structures often pass unnoticed at work and at an organizational level [24]. Inspiration from an intersectional approach was taken in order to move away from reductionist notions of gender and the risk of reproducing men and women as two separate groups with no internal differences [27, 28]. In this study, we have made the strategic choice to primarily focus on two social categories, namely gender and age [29].

The study aims to explore managers’ experience of 1) causes of sick leave in the personal and work-life of young employees with CMDs, and 2) the prevention of such sick leave. A gender perspective is applied to examine managers’ experience of the causes and prevention of sick leave in relation to male and female employees and male and female-dominated occupations.

## Method

### Study design

This study has a qualitative design which is suitable for the purpose of exploring and describing nuances in experiences and perspectives among involved stakeholders [30]. The reporting of this study was guided by the consolidated criteria described by Tong et al. [31]. The gender-theoretical framework influenced the research design (aim, method, and analysis), which was constructed so that it could capture gender- and age-related factors observed by the managers. The study is part of a research project aiming to explore, from a gender perspective, how young employees and managers perceive the causes of sick leave and the return-to-work process among young adults with CMDs [21].

### Setting and procedure

Research participants were recruited nationally in Sweden between November 2020 and August 2021 through advertising on the university homepage, in industry magazines and newsletters directed at managers, on social media sites such as LinkedIn, and by distribution of flyers. Companies and unions with which the research group already had established connections were also contacted. Twenty-nine managers emailed their interest in participating to the research group. Two were not included since they did not work as managers and three could not be reached to book an interview. Twenty-four managers agreed to be interviewed but one did not show up for the scheduled interview. To be included, participants needed to have recent experience (currently or within the years preceding the interview) in a first-line manager position (≥ 1 year, ≥ 50% of fulltime) and supervising a young employee (19–29 years) who had been on short-term sick leave (3–12 weeks) due to one or more CMDs.

### Data collection and interviews

Semi-structured interviews were conducted with 16 female managers and seven male managers between 36 and 61 years old. The characteristics of the participating managers are presented in Table 1.

**Table 1:** Managers’ characteristics.

	Women	Men
**Number**	16	7
**Age in years, mean, (range)**	48 (36–61)	50 (44–59)
**Occupational sector** *		
Health care/Social service/Education	11	1
White collar	5	2
Service	2	1
Blue collar	0	3
**Years of experience as a manager**		
≤ 5 years	3	1
6–10 years	6	1
≥ 11 years	7	5

*Two managers had experience from more than one occupational sector. These participants are represented in multiple rows.

The development and testing of the interview guide have been described in a published study protocol [21]. HTL and CO, both of whom are experienced with the semi-structured format, conducted the interviews. Each interview lasted around one hour and was held digitally using video platforms (MS Teams and Zoom). The interviews covered subjects relating to causes of sick leave, prevention, and return to work (RTW) after sick leave. In this article, the focus is on causes and prevention before the sick leave happens (for issues relating to the sick leave period and RTW, see [32]).

The guide allowed open questions about the managers’ experience of supervising young employees with CMDs, their thoughts about possible causes of sick leave and several follow-up questions about work, personal life, lifestyle, and expectations about work-life. The guide also included questions about the possibility of preventing sick leave based on the managers’ experience. Throughout the interviews, the interviewers repeatedly reminded the managers that young employees with CMDs were in focus. Managers were asked to reflect upon the possible impact of gender and young age, whether the occupation was male or female dominated, and whether any of these issues posed specific challenges to young employees or required the managers to deal with them differently for young employees than for older employees.

### Ethics

This study was approved by the Swedish Ethical Review Authority (registration number 2020–03271). All participants received written and oral information about the research project and gave informed and written consent before participating in the interview. The Swedish Research Council for Health, Working Life, and Welfare financed this project (Forte; grant number 2019–00883), but did not influence the research process.

### Data analysis

The analysis has been described in detail elsewhere [21]. In short, we used Heish and Shannon’s conventional content analysis because this method is suitable for new exploratory research, and derives its codes and categories from the empirical data [33]. Analysis began after all interviews had been conducted and transcribed verbatim. To increase the credibility of the analysis, all authors participated throughout the process. The entire research group read and discussed four interviews at an early stage in order to familiarize themselves with the data material. This initial discussion also made it possible to use the collective knowledge and experience in the research group. Two of the authors have managerial experience at various levels, one with extensive experience in that role. Several of the authors have long experience in the area of occupational medicine and have carried out several studies of sick leave due to CMDs and rehabilitation. HTL and CO read all interviews several times and jointly coded the first four interviews to reach a shared understanding of how to code the content. In particular, this process facilitated a discussion of ambiguous interview statements. After coding and discussing the first four interviews, they coded the remainder of the interviews independently but in close contact to enable discussion of potential new codes. Each interview was summarized to get a sense of the whole. HTL, CO, IJ, LN, and EBB had regular meetings to discuss and reflect upon the codes. Throughout the analysis, the research group had continuous open discussions about interpretations from different perspectives and positions relating to managerial experience, age, work-life experience, and academic discipline. As Graneheim and Lundman have argued, having more than one researcher involved in the analysis increases the dependability of the analysis [34]. The coding was performed using NVIVO software [35]. Reflective notes were taken on the managers’ experience of similarities and differences between young male and female employees and between female and male-dominated occupations. In the following analysis, all codes were examined, similar codes were grouped together to form subcategories, and similar subcategories formed main categories. The notes taken on gender differences and similarities were used in this step, and subcategories containing gender differences were marked. All authors discussed these categories until a consensus was reached. Finally, as is prescribed in the conventional content analysis approach, the study’s findings were compared and contrasted with relevant theories in the discussion section [33].

## Findings about causes of sick leave

The first section of the findings covers causes of sick leave due to CMDs, and the second covers managers’ experience of the prevention of this type of sick leave. The analysis resulted in four main categories and eight subcategories describing managers’ experience of the causes of sick leave (Table 2).

**Table 2:** Categories and subcategories covering managers’ experience of causes of sick leave for young employees with CMDs.

Subcategories	Categories
Having negative experiences in the past* Demanding, fast-paced society and educational system*	**Entering workforce when already worn-out**
Not being able to see what counts as good enough* Difficulties dealing with demands from colleagues*	**Struggling with too high expectations at work**
Problematic personal relationships or family life* Not being able to adjust life to accommodate work	**Having a challenging personal life**
Being stuck in the wrong job Having low control over the structure of the workday*	**Being unable to manage specific occupational challenges and demands**

*Gendered aspects of managers’ perceptions of differences between young male and female employees (including differing gendered expectations) or managers’ perceptions of differences between female and male-dominated occupations.

### Entering workforce when already worn-out

This category includes the causes managers observed related to the young employees’ lives preceding their entry into the workforce. According to the managers, these causes had their roots in past negative experiences or were related to earlier demands facing young adults in societal and educational contexts.

#### Having negative experiences in the past *

Managers reported that young employees’ sick leave could be related to traumatic events during childhood or having a previous history of CMD. They noted gender differences in negative experiences from the past. One manager illustrated the difference in terms of bearing an emotional burden:

Young women are more stressed about their performance while men carry emotional baggage with them. So, for example, bullying or alienation or something (IP 18, female manager, white collar/health care).

The managers had observed that young male employees with a troubled childhood or school history were still so severely affected by this that it led to sick leave when they started working life. Young female employees’ sick leave, on the other hand, was more commonly related to present events.

According to the managers, young employees who went on sick leave had often suffered from CMDs for some time before their employment:

What I experience is that I have a number of young people who are struggling with mental ill-health, and I would say that all of them have had it throughout their upbringing. This is not something that begins when you are out of your teens. It comes earlier. (IP 18, female manager, white collar/health care)

Managers also said that some young employees are more sensitive to stressors and vulnerable to life challenges than others.

#### Demanding, fast-paced society and educational system *

When managers reflected upon why young employees under their supervision had gone on sick leave, they described persons who were vulnerable and had trouble coping in a context that managers regarded as demanding and fast-paced. Managers referred to societal and educational contexts that had left little or no room for young adults to make mistakes. The educational system was a particular source of concern, and managers perceived their young employees as already drained of energy when entering the workforce:

They come pretty much run down from high school, I can tell you (IP 2, female manager, service/white collar).

One common remark was that young people have grown up in and been affected by a fast-paced society:

In general, I think it could be that it is some kind of stress factor, that you have to make a career and there are so many other things… There is so much information in society today compared to when I was young and growing up. (IP 11, male manager, white collar)

Managers also said that these tendencies have worsened over time and are reinforced through social media by exposing young people to others’ success stories. In the view of the managers, gendered expectations experienced by young women make them feel that they should be responsible persons, particularly concerning social relationships with others. Managers mentioned this social responsibility as something that added to young female employees’ overall burden, and they considered this responsibility an aggravating factor for CMDs. One manager recalled that several female employees had similar histories in this regard, and were people who:

… took on a great deal of responsibility at an early age at home, continue at school and are successful, achieve, help others and… […] I guess that is the category that I have seen, that I work with. (IP 16, female manager, social service)

### Struggling with too high expectations at work

This category includes managers’ descriptions of young employees struggling with their own and others’ expectations at work. The managers had observed that young employees could experience problems if they were not able to identify what counts as good enough or if they had difficulty dealing with demands from colleagues.

#### Not being able to see what counts as good enough *

Managers described young employees on sick leave as overly ambitious and taking on extra responsibilities very soon after taking a new job. Managers said that these early responsibilities risked inducing a negative spiral that could result in sick leave. Managers also said that young employees’ lack of experience left them unprepared for prioritizing tasks and understanding what counts as good enough:

When you are young, you do not have the same experience. You have to be introduced wisely. And not to set the bar like a 35 or 40-year-old colleague. And to also accept that it is okay to be good enough based on my experience. (IP 6, male manager, education)

Managers found that these tendencies were more common in young female employees than young male employees:

I think they [young female employees] feel they need to have a higher performance [than their male counterparts], actually. Unfortunately. I’m not quite sure why, though, or understand why. Because we do not have… We do treat, well, everyone equally, I was going to say… We do not set different standards for guys and girls in delivery. (IP 5, male manager, blue collar)

Managers emphasized that young employees often want to prove themselves and demonstrate their capability from day one. They described being a young female with high ambitions as also being associated with taking on too much responsibility too soon, working long hours, or bringing thoughts or worries about work home after finishing the working day. Examples of these tendencies could include checking e-mails after office hours or becoming the contact person for several residents at nursing homes. Managers in human service occupations emphasized that it can be challenging for their young employees to draw limits when working with people such as patients, care recipients, or pupils:

Working life can sometimes be quite tricky, sorting out what’s important and what’s less important. Well, over the years and with experience, you can sort that out better. (IP 3, female manager, social services)

#### Difficulties dealing with demands from colleagues *

Managers from female-dominated occupations reflected on the demands placed on female employees compared to male employees as a risk for sick leave:

I do not see the same risk, or I am not worried about the younger guys in our team. Because they do not have the pressure […] as I experience it. Not on themselves and not from anyone else. Because I also see that we… the workgroup is not as kind to the younger girls. They make much higher demands [of the girls] than of the guys. It is enough that the guys are nice and want to be with us, while the girls have to go in and know almost everything straight away. (IP 16, female manager, social service)

Aligned with this understanding of young employees was the thought that being young could mean having difficulty letting go of negative experiences or unfulfilled demands relating to work. Some managers related this characteristic more to gender than to age:

… men generally have an easier time letting what happens to them drain off (IP 18, female manager, white collar/health care).

This difficulty in dealing with demands at work or from colleagues was described by the managers as a cause of sick leave for young women but not for young men.

### Having a challenging personal life

This category covers causes that managers associated with the current personal situation of their young employees. This included having problematic relationships or not yet having learned to adapt their everyday routines outside of work in order to manage their work life.

#### Problematic personal relationships or family life *

Some managers had insight into young employees’ personal lives and reported causes either in their relationships with a partner or in ongoing problems in their families of origin.

I have also noticed that several of these people live in destructive relationships, both partner relationships and other destructive relationships, relatives, siblings, parents, which may not be so healthy. (IP 7, Female manager, Social services)

In the experience of the managers, problematic or non-supportive relationships in the personal lives of young employees led to feelings of stress, which managers regarded as a cause of sick leave. Examples of this were having children early in life and being a sole parent (or lacking social support in this regard). Managers reported that these factors affected their young female employees particularly:

I would say the situation in the family is messy. They might have small children, they’re divorced, they have a new family with a new guy and it’s working out so-so, and then they’re moving and so … And then it kind of falls apart for them. (IP 8, Female manager, Social services)

#### Not being able to adjust life to accommodate work

In contrast to managers who had experienced overly eager employees, some managers reflected upon young employees who were not adequately prepared for their new position, or who were unable to adjust their personal routines to accommodate work. The importance of adjustments in personal life, such as adjusting leisure-time activities (e.g., not hanging out with friends late at night), could be particularly challenging when doing shift work. Managers who supervised young, shift working adults described this as demanding. They reported that young workers need to learn to be agile and make recurring adjustments (e.g. to get enough sleep). Not making adjustments for shift work was described as something that:

…works for a few weeks or a few months, then you have to start prioritizing work. (IP 4, male manager, blue collar).

### Being unable to manage specific occupational challenges and demands

Managers had observed how a mismatch between the young employee and their work could cause or exacerbate symptoms of CMDs which could eventually lead to sick leave. Being stuck in the wrong job or finding the structure and flow of the workday difficult were examples of this type of mismatch.

#### Being stuck in the wrong job

Managers reflected that young employees who struggled to keep up with work tasks, were not adequately prepared for their new position, and were unhappy about their current work situation had problems when trying to move on. Managers said that the limited opportunities for finding new work could be a cause of sick leave in young employees:

Sometimes you might want to train for something else, but it becomes a trap. You may not have the financial means. […] But staying on even though you do not like it can be one of the things that makes you take sick leave. (IP 14, female manager, social service)

From the managers’ perspective, some young employees had gone on sick leave because their job was too demanding or harder than they had anticipated. Sectors in which it was relatively easy to get employment as a young person (e.g., occupations in the care sector that do not require specific education) stood out in this regard:

If you have a job you cannot manage, then you get sick in the long run. So it is about being capable of doing your job too. And there you might have something with young people, that they jump on jobs that are actually a bit too difficult for them. (IP 2, female manager, service/white collar)

#### Having low control over the structure of the workday *

Managers reflected upon the differing demands in female-dominated and male-dominated occupations. They observed that professions that involve work with other human beings and work in which employees have little control over the structure of the workday, could aggravate pre-existing vulnerability to mental health problems:

If you have a job where you can balance your working day a little bit more, or can balance a little bit more from one day to the next what you prioritize to work on, that you have a freer work content, then it’s a huge difference compared to… when you might come to work and feel a little bit down, and then you meet a class, which also sets something in motion. Or you meet people in severe vulnerability. (IP 23, female manager, education)

Managers observed that these work situations were more common in some female-dominated human service occupations. When asked if their occupation posed specific challenges for young employees, low control over the day-to-day work pace was a recurring example given by managers working in emergency care and other human-oriented occupations. For example, one manager compared the considerable challenges facing employees in what she termed female occupations (such as nurses and childcare workers at preschools) with the situation for builders or carpenters. In her view, workers in female-dominated occupations might have a harder time controlling parts of their workday, adding to the strain of the work situation.

## Findings about prevention of sick leave

One category and three subcategories could be identified from the analysis of managers’ experience of preventing sick leave due to CMDs, as presented in Table 3.

**Table 3:** Managers’ experience of and thoughts about prevention of sick leave for young employees with CMDs.

Subcategories	Category
Introduction, supervision, and mentorship	**Easing the transition into work life**
Communication about workers’ health and problems at work*	
Time for collegial reflection and recovery at work	

*Gendered aspects of managers’ perceptions of differences between young male and female employees (including differing gendered expectations) or managers’ perceptions of differences between female and male-dominated occupations.

### Easing the transition into work life

In the experience of the managers, if sick leave was to be prevented, young employees needed to be guided into new workplaces and work positions and into making the transition to work-life. Managers also emphasized the importance of early communication and time for collegial reflection as preventive factors. The three subcategories below give examples of how managers tried to facilitate this transition.

#### Introduction, supervision, and mentorship

Managers discussed the importance of a good introduction and continuous supervision and mentorship in preventing sick leave among young employees, for example, by individualized leadership:

So, if you bring in young people, you have to… you have to understand that in the beginning, before you can let them go completely free, you have to have a fairly close leadership and fairly individualized as well, so that you can actually control a bit. Because these people often sink their teeth into too many things at the same time, and then it becomes difficult to prioritize. So that the manager actually steps in quite clearly and says that this is the most helpful thing for the company right now. (IP 10, female manager, white collar)

As illustrated by the quote, managers saw the need to put the brakes on young, ambitious employees. Managers also saw a greater need for introduction, supervision, and mentorship for young employees to ameliorate their lack of experience and their newness in the labour market, the workplace, or the work position. Managers wished they had more time to guide the new employee and help limit their engagement. Thus, managers acknowledged the importance of continuous supervision (by the manager) or mentorship (by senior colleagues), but said that close supervision could not always be a priority when the overall workload at the workplace became too heavy. Managers with experience in human service occupations described young employees as more fragile when new at work. Young employees did not always know yet:

…how deep one could go into working with patients before getting hurt (IP 17, female manager, health care).

Supervision by more senior personnel was seen as crucial, but not always feasible due to a lack of resources.

They have not ended up with mental ill-health yet, but I see the risk that they will. They come right out of high school, they have had internships with us, they are so amazing in every way, and they go in and get and take a responsibility that they cannot even handle. We have said it is not healthy to do that initially, but that even if they want to and can, the rest of us can’t give it to them. But more just reinforce to them that they are good […] but they have to get into the other work before they take on too much. (IP 16, female manager, social service)

Managers highlighted the importance of continuous support when giving young employees more responsibility, new roles, or assignments at work. For example, one manager said that what he had learned in retrospect from supervising a young woman on sick leave was the need to follow up and evaluate any transitions into new work roles carefully.

#### Communication about workers’ health and problems at work *

This subcategory includes managers’ observations that their ability to work preventively depended on good communication with young employees. One factor that could interfere with managers’ ability to communicate was their own high workload. Managers also said that communication was affected by the fact that many young employees seemed to feel the need to prove themselves at work and were therefore unwilling to discuss problems with their manager.

Unfortunately, talking about mental health is a bit of a taboo for many people. But when you can see that they have progressed a bit and can talk openly about it, then you can see that it helps these people so much when they can be a bit open about their mental health. (IP 3, female manager, social services)

Managers also reflected upon observed gender differences, saying that young female employees were generally keener to prove their capabilities at work, which made them less eager to signal to the manager that they were struggling. In contrast, other managers said that young female employees were better at talking about their mental health status than young male employees:

Because I think… or know that guys feel just as bad as girls, but we have a culture for girls to talk about it. But guys do not (IP 16, female manager, social service).

In retrospect, managers wished their employees had come to them before sick leave to discuss their feelings and work capacity problems. Managers also reflected upon the shared responsibility of both the manager and the young employees to communicate when work became too complex or too intense, in order to give the manager a chance to help with support and prioritization:

And then it is a failure on the employer’s part, of course, that we have not been able to pick up on these signals. But I would also say that the employee themself may not have taken full responsibility for their own situation. I expect a 40-hour working week from all my employees, that’s all. And if you don’t manage to get your work done in those 40 hours, I help you prioritize. (IP 10, female manager, white collar)

Managers worked actively and in different ways to create a workplace atmosphere that facilitated communication. For example, they reported using various strategies, such as trying to create a psychologically safe climate at work by carefully asking about previous experiences of rough periods in life in order to adjust demands at work from the beginning. Managers also said that they continuously discussed work-life balance and acknowledged that everyone could sometimes experience hardship.

Well, it is not, it is not just work. It is the whole thing. It is this life puzzle, maybe, and also… I definitely think it is a combination of the individual’s characteristics, how you are as a person, and then what you have outside of work, so the life puzzle, the life situation and the situation at work of course. And it is the situation at work that I can influence. (IP 5, male manager, blue collar)

Managers also said that having a variety of communication strategies for measuring overall worker health was an essential aspect of their preventive work. For example, they had recurring one-on-one conversations with employees, followed up on occasional sick days, or used tools where employees reported their current stress levels or work imbalance. From the managers’ perspective, these tools could make catching early signs of CMD easier when young employees might find communication challenging.

#### Time for collegial reflection and recovery at work

When managers reflected actively on their responsibilities for worker health and preventing sick leave among young employees, they emphasized the benefits of having a good and open social work environment. In this subcategory, managers did not mention differences between young male and female employees. Instead, they reported that they worked preventively by creating a workplace where one could be open about symptoms of CMDs and reduced work capacity, which was regarded as beneficial for young employees regardless of gender. One manager took her own divorce as an example of how going through a challenging experience had increased her understanding that rough times could happen to anyone during the course of a working life. In her experience, her employees had referred to the example she set as a manager and had found comfort in the fact that it was a workplace where one could find understanding and support if needed. Managers who knew that young employees had problems in their personal lives strong enough to cause CMDs said that even though they might not be able to help them with the core problem, they could at least:

…start to assess the situation at work, and the demands of the job, so that one lower the expectations at work during that period (IP 23, female manager, education).

In addition to temporarily lowering expectations for struggling employees, managers highlighted the preventive function of time for collegial reflection, especially for new and young employees. One manager regarded facilitating a sustainable work life as one of her most important responsibilities as a manager, and exemplified how she put this into practice:

And then my other primary task at work is to make sure that staff can feel good so that they have a sustainable working life. […]…we try to work with reflection, breaks, and recovery. (IP 14, female manager, social services)

## Discussion

This paper aimed to explore managers’ experience of causes and prevention of sick leave due to CMDs among young employees. A gender perspective was applied to examine this in relation to male and female employees in male- and female-dominated occupations. In terms of *causes of sick leave* in young employees, managers identified entering the workforce already worn-out; struggling with too high expectations at work; having a challenging personal life; and being unable to manage specific occupational challenges or demands. These causes were related both to the employees’ work- and private life and were at times regarded as intertwined by the managers. In terms of *preventing sick leave* due to CMDs for young employees, managers identified the importance of easing the transition into working life.

### Gendered aspects of causes of sick leave

In this study, managers had observed gender aspects in causes of sick leave and related these to differences between female and male dominated occupations and between young male and female employees. In this regard, they echo the two hypotheses commonly used to explain gender differences in sick leave [11]. In observing that female-dominated and human-oriented occupations involve specific risks, managers were close to the “exposure hypothesis”. Their observations that young female employees are more burdened by demands than young male employees are in line with the “vulnerability hypothesis.”

Our findings revealed the managers’ perceptions of young female employees as ambitious and burdened by work demands. The finding aligns with previous studies which have found that women [36] and young women in particular [37, 38] perceived that the high demands made of them played a part in their going on sick leave. Limiting their workload was not seen as a solution because of demands made by employers and managers [38]. The results in our study add that managers also recognized that young employees, and young female employees in particular, felt burdened by high expectations and regarded this as a cause of sick leave. A previous study has concluded that Swedish girls as young as age 17 already report high levels of stress and that demands made of oneself are a common cause of this stress [22]. That young female employees struggle with high demands was also reported by managers with experience of supervising female employees in male-dominated occupations in our study. However, managers with experience of female-dominated occupations reflected on the intersecting categories of age and gender, with more pressure on young female than young male employees. These managers observed in addition that gendered and lower expectations were made of young males in female-dominated, human-oriented occupations organizations. According to their understanding, young male employees were highly valued at the workplace, which meant that they were not exposed to the same risk of pressure leading to sick leave as young female employees. In addition, managers viewed human-oriented and commonly female-dominated sectors, such as education and health care, as particularly challenging for young employees struggling with emerging symptoms of CMDs. Managers found that it was sometimes difficult, in human-oriented occupations, for an employee to influence the work pace or draw boundaries. This tendency not to draw limits and to put others’ needs (e.g., care recipients) before one’s own has been termed “compulsive sensitivity” and has been linked to norms of femininity and care at work and in personal life [39, 40].

### Gendered aspects in the prevention of sick leave

When managers in our study reflected upon preventive work and young employees, they emphasized that communication should be initiated early on by the employee, but they also reported gender differences behind the unwillingness to communicate. Focusing on an employee’s responsibility to be transparent and open when struggling at work due to a CMD is also symptomatic of the managers’ view of sick leave as an individual problem rather than an organizational one. This result aligns with a previous study showing that managers focus on causes and interventions at an individual level rather than at organizational level [41]. Ladegaard et al. [41] argued that emphasizing individual characteristics and situations could ease managers’ feelings of responsibility and sometimes guilt, but could also weaken intentions to introduce organizational changes [41]. It has previously been argued that increased gender sensitiveness, on multiple levels, is crucial in systematic occupational safety and health management [40]. In our study, all managers, on the basis of their experience, were eager to try to prevent sick leave. Despite their efforts though, it was evident that they had a rather individualistic view of CMDs. One conclusion from our study is that it could be beneficial to combine a more organisationally oriented approach with increased awareness of work-related gender norms and expectations, in order to be able to identify risk situations and initiate preventive actions.

Perceived stigma connected to CMDs is a well-known obstacle to communication [41, 42], which the managers in our study reflected, especially pertaining to young male employees. Other studies have argued that men’s reluctance to talk about CMDs might make it more difficult for managers to become aware of CMDs in their male employees [42]. In the experience of the managers in our study, the situation of young female employees was more complex: while they could talk more openly about mental health issues than male employees, their eagerness to prove themselves at work hindered them from asking for help and bringing up work problems with their manager in time. In order to facilitate communication about both mental health issues and work-related problems, managers used themselves and their own experiences to set an example and normalize occasional difficulties that could affect work and performance.

In our comprehensive project (of which this study is a part), we conducted interviews with young employees. A notable difference between the perceptions of the young employees and the perceptions of the managers was that the employees reflected upon how their insecure position in the labour market stopped them from communicating problems at work with their managers [20]. In contrast, the managers interviewed here did not mention insecure employment as an issue with regard to managing CMD at the workplace. The risk of losing one’s position or employment has been identified in previous research as a hindrance to communication [20, 43]. Precarious and temporary employment has been associated with mental health issues [44], particularly for young employees [7]. However, only a few studies have incorporated a gender perspective of precarious employment and mental health [45]. Future research into preventive actions and communication about CMD could help clarify gendered and age-related factors pertaining to insecurity and precarious employment.

#### Strengths and limitations

In this qualitative study we strove to increase the credibility and transferability of the results [46] by describing our research process in detail and by triangulation of researchers, all identifying as women but at different career stages, and from several disciplines, including occupational health, occupational medicine, gender studies, and social science. The aim of the study was to provide insights into managers’ experience of the causes and prevention of sick leave due to CMDs among young adults. One strength of the study is that we included managers from both blue- and white-collar sectors with experience of at least one young employee who had become sick-listed due to CMDs. We also interviewed managers with various types of managerial experience. The great majority (19 out of 23) had been a manager for six years or longer and could thus contrast and compare their more recent experiences with earlier ones. A qualitative method was chosen in order to provide in-depth knowledge, since the managers were able to describe their experiences with their own words. A possible limitation of the study is that the respondents might have had recall bias [47]. Another limitation is that respondents were recruited through advertising, and it is possible that managers who were interested in and open-minded about CMDs were more likely to volunteer. There could also be a risk of social response bias since the managers were responsible for the organisational and social work environment.

## Conclusion

This study contributes new knowledge about perceived risk situations and contexts by examining managers’ experience of supervising young male and female employees with CMDs. It highlights the need to ease the transition into working life for young employees and the need for a supportive and detailed introduction to work, with continuous supervision and frequent communication at both individual and workplace levels as preventive actions. The managers’ experience demonstrates that gender norms and expectations are factors of importance in the development and prevention of CMDs. Our findings emphasize the importance of taking gender into account in communicating about work demands and the occasional need to reduce demands to prevent sick leave due to CMDs, regardless of whether the employee is facing challenges at work or in their personal life.
